# Differential gene expression profiling of endometrium during the mid-luteal phase of the estrous cycle between a repeat breeder (RB) and non-RB cows

**DOI:** 10.1186/s12958-017-0237-6

**Published:** 2017-03-23

**Authors:** Ken-Go Hayashi, Misa Hosoe, Keiichiro Kizaki, Shiori Fujii, Hiroko Kanahara, Toru Takahashi, Ryosuke Sakumoto

**Affiliations:** 1 Division of Animal Breeding and Reproduction Research, Institute of Livestock and Grassland Science, National Agriculture and Food Research Organization, Tsukuba, 305-0901 Japan; 2 Division of Animal Sciences, Institute of Agrobiological Sciences, National Agriculture and Food Research Organization, Tsukuba, 305-8602 Japan; 3 Cooperative Department of Veterinary Medicine, Faculty of Agriculture, Iwate University, Morioka, 020-8550 Japan

**Keywords:** Repeat breeder, Endometrium, Caruncle, Intercaruncle, Microarray, Cow

## Abstract

**Background:**

Repeat breeding directly affects reproductive efficiency in cattle due to an increase in services per conception and calving interval. This study aimed to investigate whether changes in endometrial gene expression profile are involved in repeat breeding in cows. Differential gene expression profiles of the endometrium were investigated during the mid-luteal phase of the estrous cycle between repeat breeder (RB) and non-RB cows using microarray analysis.

**Methods:**

The caruncular (CAR) and intercaruncular (ICAR) endometrium of both ipsilateral and contralateral uterine horns to the corpus luteum were collected from RB (inseminated at least three times but not pregnant) and non-RB cows on Day 15 of the estrous cycle (4 cows/group). Global gene expression profiles of these endometrial samples were analyzed with a 15 K custom-made oligo-microarray for cattle. Immunohistochemistry was performed to investigate the cellular localization of proteins of three identified transcripts in the endometrium.

**Results:**

Microarray analysis revealed that 405 and 397 genes were differentially expressed in the CAR and ICAR of the ipsilateral uterine horn of RB, respectively when compared with non-RB cows. In the contralateral uterine horn, 443 and 257 differentially expressed genes were identified in the CAR and ICAR of RB, respectively when compared with non-RB cows. Gene ontology analysis revealed that genes involved in development and morphogenesis were mainly up-regulated in the CAR of RB cows. In the ICAR of both the ipsilateral and contralateral uterine horns, genes related to the metabolic process were predominantly enriched in the RB cows when compared with non-RB cows. In the analysis of the whole uterus (combining the data above four endometrial compartments), RB cows showed up-regulation of 37 genes including *PRSS2*, *GSTA3* and *PIPOX* and down-regulation of 39 genes including *CHGA*, *KRT35* and *THBS4* when compared with non-RB cows. Immunohistochemistry revealed that CHGA, GSTA3 and PRSS2 proteins were localized in luminal and glandular epithelial cells and stroma of the endometrium.

**Conclusion:**

The present study showed that endometrial gene expression profiles are different between RB and non-RB cows. The identified candidate endometrial genes and functions in each endometrial compartment may contribute to bovine reproductive performance.

**Electronic supplementary material:**

The online version of this article (doi:10.1186/s12958-017-0237-6) contains supplementary material, which is available to authorized users.

## Background

Repeat breeder (RB) is generally defined as any cow that has failed to conceive after at least three inseminations. In both dairy and beef cattle herds, the presence of RB cows can directly lead a large economic loss for producers due to an extension of the length of the open period and frequent artificial insemination (AI) [1]. In addition to management problems such as inadequate estrus detection and AI techniques, various physiological problems of individual cows are one of major causes of repeat breeding. For example, infections of uterus, cervix and/or vagina, dysfunctions of uterus or ovary, obstructed oviducts, defective oocytes and anatomical defects of the reproductive tracts are involved in conception failure, early embryonic death and endocrine disorders of RB animals. [1]. It has been reported that embryo transfer is effective to improve the fertility of RB cows and heifers [2, 3]. On the other hand, a study of reciprocal transfers of embryos between RB and virgin heifers showed that a higher proportion of embryos transferred from RB to virgin heifers than from virgin to RB heifers survived at day 16 to 17, suggesting that the uterine environment in RB heifers is less suitable than in the virgins for supporting a successful embryo development [4]. This became more evident by transfer of identical demi-embryos to RB and virgin recipient heifers resulted less number of morphologically normal and elongated embryos in the RB heifers than in the virgin heifers at day 15 [5]. About an association between alteration of uterine environment and repeat breeding, Katagiri et al. have demonstrated that there is a close relationship between the endometrial epidermal growth factor profile and diminished fertility of RB cows [6].

The molecular mechanisms underlying endometrial function may contribute to reproductive performance in cattle. Increasing evidence using global gene expression analysis has identified numerous differentially expressed genes and related functional pathways in bovine endometrium among highly fertile, subfertile and infertile animal strains during estrous cycle or early pregnancy [7–10]. Recent studies have also investigated gene expression profiles under various conditions of the bovine endometrium during the estrous cycle and/or during early pregnancy using DNA microarray or RNA sequencing [11–18]. In addition, microarray studies have revealed that heat stress and steroid hormones directly affect bovine endometrial gene expression profiles [19, 20].

In ruminants, the endometrium shows structural and physiological differences depending on the uterine compartments. The caruncular (CAR) areas are aglandular and a limited area that forms placentomes by fusing with the fetal extraembryonic membrane [21, 22]. On the other hand, the intercaruncular (ICAR) areas contain endometrial glands that synthesize and secrete substances or factors that are essential for survival and development of the conceptus [23, 24]. A study that directly compared the gene expression profiles of CAR and ICAR during implantation in cows showed 1177 and 453 differentially expressed genes (DEG) were found for cyclic and pregnant animals, respectively [13]. In addition, it has been reported that tissues of the ipsilateral uterine horn to the ovary with the corpus luteum (CL) contain greater quantities of progesterone (P4) and are more sensitive to P4 as compared with tissues on the contralateral side [25]. Although a previous study demonstrated that a few genes show differences in expression between ipsilateral and contralateral uterine horns during the bovine estrous cycle [11], we consider that it is important to analyze each compartment of the bovine endometrium separately in order to understand enodometrial function more comprehensively.

These previous studies suggest that alteration of the endometrial function due to changes in gene expression may contribute to their lower reproductive performance in RB cows, whereas details of the molecular mechanisms and biological pathways of their endometria still need to be elucidated. Thus, we hypothesized that there is a characteristic gene expression profile in the endometrium of the RB cows. This study aimed to investigate differences in gene expression profiles of the endometrium between RB and non-RB cows during the mid-luteal phase of the estrous cycle. In pregnant cattle, maternal recognition of pregnancy occurs around Day 14–15 [26]. In addition, it has been reported that the majority of early embryo losses in cattle have occurred within 16 days of gestation (i.e. during the mid-luteal phase) [27, 28]. Therefore, the basal gene expression profiles of endometrium at mid luteal phase would have the most important association with reproductive performance.

## Methods

### Animals and sample collection

This study was carried out using non-lactating Japanese Black cows at the institute’s ranch (age: 7.8 ± 0.9 years, parity: 3.3 ± 0.8, open period from last parturition to first AI in this study: 104 ± 9.6 month). Repeat breeder cows (*n* = 4) were defined based on a previous study by Dochi et al. [3]. Briefly, the RB cows had three characteristics as follows: (1) detectable estrous behavior, but not always normal estrous cycles; (2) not conceiving after three or more inseminations following normal estrous behavior; and (3) healthy uterus and ovaries, as determined by transrectal palpation. Non-RB cows (*n* = 4) conceived within three inseminations. The non-RB cows were confirmed to be pregnant by transrectal ultrasonography (HS-1500V; Honda Electronics. Co., Aichi, Japan) at 40 days after insemination, then abortion was induced by a single intramuscular injection of 500 μg of prostaglandin F2α (cloprostenol [Dalmazin]; Kyoritsu Seiyaku. Co., Tokyo, Japan) followed by repeated normal estrous cycles at least twice. Both RB and non-RB cows were slaughtered on Day 15 of the estrous cycle (the day of estrus was designated as Day 0) and the uterus and both ovaries together were collected. Uterine horns were identified as ipsilateral to the ovary containing the CL or contralateral. We collected CAR and ICAR in the endometrium from the middle area of each uterine horns. The uterine horns were cut opened longitudinally using scissors and CAR were carefully dissected first not to include ICAR, subsequently, ICAR areas were cut off. Collected samples were snap-frozen in liquid nitrogen and stored at −80 °C until RNA extraction. Whole cross section of the uterus for immunohistochemistry were collected from the middle area of ipsilateral uterine horn of all cows and fixed in 10% formalin (v/v), embedded in paraffin wax, and then stored at 4 °C until use. All procedures in animal experiments were carried out in accordance with guidelines approved by the Animal Ethics Committee of the National Institute of Agrobiological Sciences for the use of animals (permission number: H18-036).

### Microarray analysis

Total RNA was extracted from each sample by acid guanidinium thiocyanate-phenol-chloroform with ISOGEN (Nippon Gene, Tokyo, Japan) according to the manufacturer’s instructions. All RNA samples were then treated with TURBO DNase (TURBO DNA-free™ Kit, Thermo Fisher Scientific, Waltham, MA, USA) according to the manufacturer’s instructions to remove contaminating genomic DNA. The quantity and quality of the total RNA samples were assessed using a NanoDrop spectrophotometer (ND-1000; NanoDrop Technology Inc., Wilmington, DE, USA) and an Experion automated electrophoresis system with an Experion RNA StdSens kit (Bio-Rad Laboratories, Hercules, CA, USA), respectively. A custom-made bovine oligonucleotide microarray with 15,000 unique genes (GPL9284) fabricated by Agilent Technologies (Santa Clara, CA, USA) was used in this study, which was performed as described previously [29]. Sixty-mer nucleotide probes for the customized microarray were synthesized on a glass slide. We performed one-color microarray analysis. cDNA synthesis, Cy3-labeled cRNA preparation, hybridization, and the washing and scanning of array slides were performed according to the Agilent one color microarray-based gene expression analysis protocol. Briefly, 400 ng of total RNA from each sample were reverse-transcribed into cDNA using the Quick Amp Labeling Kit (Agilent Technologies) with an oligo dT-based primer, and then Cy3-labelled cRNA was prepared by in vitro transcription. Labeled cRNA was purified with an RNeasy Mini Kit (Qiagen, Hilden, Germany), and the concentration and Cy3 dye incorporation (pmol Cy3/μg cRNA) were measured with a spectrophotometer. Labeled cRNA (600 ng) was fragmented and hybridized using the Gene Expression Hybridization Kit (Agilent Technologies), according to the manufacturer’s instructions. The arrays were washed using a Gene Expression Wash Pack Kit (Agilent Technologies) and scanned using an Agilent Microarray Scanner. Feature Extraction ver. 9.5 was used for image analysis and data extraction. Microarray data from each sample were imported into GeneSpring 12 (Agilent Technologies) for further data characterization. The GEO accession numbers are as follows. Platform: GPL9284; samples: GSM2093338 to GSM2093369; series: GSE79367. To identify putative biological functions of DEG between RB and non-RB cows in each endometrial compartment, we performed functional annotation chart analysis of the lists of DEG using the Database for Annotation, Visualization and Integrated Discovery (DAVID; http://david.abcc.ncifcrf.gov/) based on Genebank Accession IDs [30]. Gene Ontology (GO) Biological Process was selected as the functional annotation category for the analysis with the threshold for minimum gene counts belonging to an annotation term set to 5 and an EASE score set to 0.05. The GO terms were ranked according to their *P*-values describing the significance of gene-term enrichment.

### Quantitative real-time RT-PCR analysis

To validate the results of microarray analysis, we confirmed mRNA expression of the following representative genes using quantitative real-time RT-PCR (qPCR) analysis: (1) top two up- or down-regulated known genes in each endometrial compartment; and (2) top five up- or down-regulated known genes in the whole uterus. Details of the procedures for single-strand cDNA synthesis and qPCR were previously described [31]. Briefly, 50 ng of total RNA from the same sample used for the microarray were reverse-transcribed into cDNA for 30 min at 48 °C using MultiScribe^TM^ Reverse Transcriptase (Applied Biosystems, Foster City, CA, USA) with a random primer, dNTP mixture, MgCl_2_ and RNase inhibitor. After heat inactivation of the reverse transcriptase for 5 min at 95 °C, PCR and resulting relative increase in reporter fluorescent dye emission were monitored in real time using an Mx3000P qPCR system (Agilent Technologies). Primers were designed using Primer Express computer software program (Applied Biosystems) or Primer3 Plus software (www.bioinformatics.nl/primer3plus/) based on the bovine sequences. The primer sequences for each gene are listed in Table 1. Thermal-cycling conditions included an initial sample incubation at 50 °C for 2 min and at 95 °C for 10 min, followed by 40 cycles at 95 °C for 15 s and at 60 °C for 1 min. The cycle threshold value (C_T_) indicate the quantity of the target gene in each sample. The relative difference in initial amount of each mRNA species (or cDNA) was determined by comparing the C_T_ values. The standard curves for each gene were generated by serially diluting plasmids containing cDNA of each individual gene to quantify the mRNA concentrations. We confirmed the utility of the dissociation curve for detecting the SYBR Green-based objective amplicon because SYBR Green also detects double-stranded DNA including Primer dimers, contaminating DNA and PCR products from misannealed primers. Non-specific amplicons appear as a peak separate from the desired amplicon peak. The expression ratio of each gene to *SUZ12* mRNA, which has been demonstrated to be suitable for normalization in bovine endometrial tissue [32], was calculated to adjust for any variations in the qPCR reaction.

**Table 1:** Details of the primers used for quantitative real-time RT-PCR analysis

Gene (GenBank accession number)	Primer	Sequence	Position
*CHGA*	Forward	5′-GCCGAAAGAGGTGACAGAAGA-3′	538-558
(NM_181005)	Reverse	5′-GTCTCCGTCCGAGTCTTCATC-3′	637-617
*CNGA1*	Forward	5′-AGCAGAGATCGCCATCAATGT-3′	1574-1594
(NM_174278)	Reverse	5′-ACCAACTCCACCAACAGACCA-3′	1663-1643
*CPXM2*	Forward	5′- ACCAGTGGATTGAAGTGGACG-3′	581-601
(NM_001206057)	Reverse	5′- TCACTCAGCCAGAGTGAGTTCCT-3′	665-643
*FAM83D*	Forward	5′- GGCTCCTACAGTTTTACATGGACAG-3′	788-812
(NM_001083393)	Reverse	5′-CAACCACTTGGCCAGACAGAA-3′	863-843
*FMO2*	Forward	5′- AAGCCAGACATCCTTTCTCTCTTG -3′	1459-1482
(NM_001163274)	Reverse	5′- CCCAACCAGGCGATACTGATA-3′	1554-1532
*GSTA3*	Forward	5′-AGAGCCATCCTCAGCTACCTTG-3′	254-275
(NM_001077112)	Reverse	5′-TCGATCCTGACTGTCTCCTTCA-3′	327-306
*IFIH1*	Forward	5′-GGGACTAACAGCTTCACCAGGT-3′	1764-1785
(XM_002685338)	Reverse	5′-GGTAACTGCATCAAGATTGGCA-3′	1860-1839
*IGG1C*	Forward	5′-ACCAAGGTGGACAAGGCTGTT-3′	274-294
(S82409)	Reverse	5′-GGAAGATGAAGACAGAGGGTCCT-3′	370-348
*KCNA2*	Forward	5′-TGGGTTCCCTATGTGCAATTG-3′	1644-1664
(NM_001101195)	Reverse	5′-TCCCGGTGGTAGAAGTAGTTGAA-3′	1734-1712
*KLHL24*	Forward	5′- TTATTGGCAAGGAGGAGATGGT-3′	901-922
(NM_001206196)	Reverse	5′- TCTCAGATCAACAGCGCGAT-3′	968-949
*KRT35*	Forward	5′- GAGACCGAGGTATCCATGCG-3′	587-606
(NM_001076073)	Reverse	5′- TTCTTGAGGCAGAGCAGCTC -3′	726-707
*LPLUNC1*	Forward	5′- TCGGTGTGTTCAACCCTAAGC-3′	1280-1300
(NM_174697)	Reverse	5′- TTCTCGTTTGGCAGCAGGAT -3′	1355-1336
*PIPOX*	Forward	5′- ACAGCATTAACACCGAGTCGG-3′	2140-2160
(NM_001014878)	Reverse	5′- GGCAGTTATGAGCCTGTTTCCT-3′	2210-2189
*PLEKHA5*	Forward	5′- GATGGATTCAAGAACGGAACG-3′	2655-2675
(XM_002687754)	Reverse	5′- TTCCACAGTCATCCTAGGTCGA-3′	2739-2718
*PRF1*	Forward	5′-CAAGCCAAATGCTAATGTCCGT-3′	408-429
(NM_001143735)	Reverse	5′-AAAGCGACACTCCACTAAGTCCAT-3′	531-508
*PRSS2*	Forward	5′-GTGAGGCTGGGAGAATACAACA-3′	211-232
(NM_174690)	Reverse	5′-ATGATCTTGGACGCATCGATGA-3′	281-260
*SLC39A2*	Forward	5′- TTGGCTGCCTATTTGCCCT-3′	355-373
(NM_001205648)	Reverse	5′- CTGGAACCACTTGAAGCAGATG-3′	428-407
*THBS4*	Forward	5′- CACTCTGAACGAGCTCTACGTGAT 3′	331-354
(NM_001034728)	Reverse	5′- GAAGAGTAAAGGCCGAAGATGGT-3′	411-389
*SUZ12*	Forward	5′-GAACACCTATCACACACATTCTTGT-3′	1565-1589
(NM_001205587)	Reverse	5′-TAGAGGCGGTTGTGTCCACT-3′	1694-1675

### Immunohistochemistry

Immunohistochemistry for chromogranin A (CHGA), glutathione *S*-transferase A3 (GSTA3) and trypsin 2 (PRSS2) was performed in the endometrium of both RB and non-RB cows on Day 15 of the estrous cycle using the automated Ventana HX System Discovery with a DabMapKit (Roche Diagnostics, Basel, Switzerland) as described previously in detail by our laboratory [33]. Uterine cross sections 7-μm-thick were incubated at room temperature with rabbit polyclonal anti-human CHGA antibody (1.0 mg/ml, 20085, ImmunoStar Inc., Hudson, WI, USA), rabbit polyclonal anti-human GSTA3 antibody (0.5 mg/ml, orb5362, Biorbyt LLC, San Francisco, CA, USA) or rabbit polyclonal anti-bovine PRSS2 antibody (10 mg/ml, OASA07087, Aviva Systems Biology, San Diego, CA, USA) diluted 1:100 (anti-CHGA), 1:20 (anti-GSTA3) or 1:200 (anti-PRSS2) in Discovery Ab diluents (Roche) for 12 h. The signals were detected using anti-rabbit IgG-Biotin conjugate (Sigma) diluted 1:500 for 1 h. Negative controls were performed using normal rabbit IgG (0.5 mg/ml, 20304, Imgenex, San Diego, CA, USA) diluted at concentrations equivalent to the primary antibodies. The sections were observed with a Leica DMRE HC microscope (Leica Microsystems, Wetzlar, Germany) and a Nikon Digital Sight DS-Fi1-L2 (Nikon Instruments Co., Tokyo, Japan).

### Statistical analysis

Microarray data were analyzed statistically with an unpaired Student’s *t*-test and summarized using GeneSpring 12 (Agilent Technologies). The analysis of each uterine compartment was performed by comparing the gene datasets which composed by microarray data of four cows in each RB and non-RB group (*n* = 4/group). The analysis of whole uterus was performed by comparing the gene datasets which composed by microarray data of all four compartments of four cows in each RB and non-RB group (*n* = 16/group). The qPCR results were analyzed using a Mann–Whitney *U* test. Results are presented as the mean ± SEM. Statistical significance is considered to be at *P* < 0.05.

## Results

### Gene expression profiles of CAR and ICAR in ipsilateral uterine horns

A total of 405 and 397 genes were differentially expressed in CAR and ICAR of the ipsilateral uterine horn of RB cows, respectively when compared with non-RB cows (adjusted *P*-value <0.05, fold-change >1.0). All data of individual gene changes in CAR and ICAR are available in Additional file 1: Tables S1 and S2, respectively. Out of these, 128 genes were up-regulated and 277 genes were down-regulated in CAR, whereas 169 genes were up-regulated and 228 genes were down-regulated in ICAR. The top 10 up- and down-regulated known genes in CAR are shown in Table 2. The most pronounced up- and down-regulation of gene expression in RB cows was observed for *GSTA3* (Glutathione *S*-transferase, alpha 3; 19.2-fold) and *CPXM2* (Carboxypeptidase X (M14 family), member 2; 5.3-fold), respectively. The top five functional annotations of DEG in the CAR of ipsilateral uterine horns between RB and non-RB cows are listed in Table 3. The GO terms involved in anatomical structure development, developmental process, cellular process, multicellular organismal development and biosynthetic process were highly enriched in up-regulated genes, whereas the GO terms involved in cellular process, cytoskeleton organization, biological adhesion, cell adhesion and cellular component organization were highly enriched in down-regulated genes.

**Table 2:** Top 10 up- and down-regulated known genes in CAR of ipsilateral uterine horns of RB cows

GenBank accession ID	Gene symbol	Gene description	Fold change	*P*-value
Up-regulated genes				
NM_001077112	GSTA3	Glutathione S-transferase, alpha 3	19.2	0.0016
NM_001206196	KLHL24	Kelch-like 24 (Drosophila)	3.0	0.0273
XM_588022	SPOPL	Speckle-type POZ protein-like	2.8	0.0239
NM_001103317	ERCC2	Excision repair cross-complementing rodent repair deficiency, complementation group 2	2.5	0.0437
XM_002696037	CD300LG	CD300 molecule-like family member g	2.2	0.0378
NM_001075908	STK33	Serine/threonine kinase 33	2.1	0.0351
NM_174607	SLC5A3	Solute carrier family 5 (inositol transporters), member 3	2.0	0.0126
NM_001192523	KCNMB4	Potassium large conductance calcium-activated channel, subfamily M, beta member 4	2.0	0.0307
NM_001083638	MEF2A	Myocyte enhancer factor 2A	2.0	0.0290
XM_002695445	ZNF211	Zinc finger protein 211	2.0	0.0063
Down-regulated genes				
NM_001206057	CPXM2	Carboxypeptidase X (M14 family), member 2	5.3	0.0496
NM_001076073	KRT35	Keratin 35	4.1	0.0279
NM_001101239	GRP	Gastrin-releasing peptide	3.6	0.0319
NM_001245926	FGF9	Fibroblast growth factor 9	3.5	0.0066
NM_174145	PKP1	Plakophilin 1 (ectodermal dysplasia/skin fragility syndrome)	2.9	0.0021
NM_001076864	TMEM129	Transmembrane protein 129	2.6	0.0087
NM_001105478	SSLP1	Secreted seminal-vesicle Ly-6 protein 1	2.5	0.0474
NM_001077962	STAC	SH3 and cysteine rich domain	2.4	0.0157
NM_001077945	PFN3	Profilin 3	2.4	0.0106
NM_001012685	FCAR	Fc fragment of IgA, receptor for	2.3	0.0322

**Table 3:** Top 5 functional annotations of up- and down-regulated genes in CAR of ipsilateral uterine horns

Term	Count	*P*-value
Up-regulated genes		
GO:0048856 ~ anatomical structure development	11	0.0029
GO:0032502 ~ developmental process	11	0.0161
GO:0009987 ~ cellular process	31	0.0186
GO:0007275 ~ multicellular organismal development	10	0.0230
GO:0009888 ~ tissue development	5	0.0246
Down-regulated genes		
GO:0009987 ~ cellular process	95	<0.0001
GO:0007010 ~ cytoskeleton organization	8	0.0061
GO:0022610 ~ biological adhesion	11	0.0065
GO:0007155 ~ cell adhesion	11	0.0065
GO:0016043 ~ cellular component organization	23	0.0099

The top 10 up- and down-regulated known genes in ICAR are shown in Table 4. The highest increase and decrease in gene expression in RB cows were observed in *LPLUNC1* (Von Ebner minor salivary gland protein; 3.7-fold) and *THBS4* (Thrombospondin 4; 3.4-fold), respectively. Table 5 summarizes the top five functional annotations of DEG in ICAR between RB and non-RB cows. As a result of DAVID analysis, only four GO terms related to metabolic process, cellular metabolic process, cellular biosynthetic process and chemical homeostasis were identified in up-regulated genes. In down-regulated genes, the GO terms involved in metabolic process, cellular metabolic process, cellular process, primary metabolic process and protein metabolic process were highly enriched.

**Table 4:** Top 10 up- and down-regulated known genes in ICAR of ipsilateral uterine horns of RB cows

GenBank accession ID	Gene symbol	Gene description	Fold change	*P*-value
Up-regulated genes				
NM_174697	LPLUNC1	Von Ebner minor salivary gland protein	3.7	0.0214
NM_001075162	FMO2	Flavin containing monooxygenase 2 (non-functional)	3.3	0.0348
NM_001166616	C5	Complement component 5	3.2	0.0429
XM_002692160	FOXA2	Forkhead box A2	3.0	0.0350
NM_181027	AKR1C4	Aldo-keto reductase family 1, member C4 (chlordecone reductase; 3-alpha hydroxysteroid dehydrogenase, type I; dihydrodiol dehydrogenase 4)	2.9	0.0104
NM_001045878	GATM	Glycine amidinotransferase (L-arginine:glycine amidinotransferase)	2.8	0.0472
NM_001206196	KLHL24	Kelch-like 24 (Drosophila)	2.6	0.0301
NM_001034419	HPGD	Hydroxyprostaglandin dehydrogenase 15-(NAD)	2.6	0.0293
XM_001254052	ZNED1	DNA-directed RNA polymerase I subunit RPA12-like	2.4	0.0476
NM_001038096	CFI	Complement factor I	2.4	0.0096
Down-regulated genes				
NM_001034728	THBS4	Thrombospondin 4	3.4	0.0106
NM_001083393	FAM83D	Protein FAM83D	2.6	0.0011
NM_001105411	GFRA1	GDNF family receptor alpha 1	2.4	0.0391
NM_001206057	CPXM2	Carboxypeptidase X (M14 family), member 2	2.3	0.0231
NM_178572	CA2	Carbonic anhydrase II	2.3	0.0474
NM_001099381	GALK1	Galactokinase 1	2.1	0.0466
NM_001035050	VTN	Vitronectin	2.0	0.0464
NM_174745	MMP2	Matrix metallopeptidase 2 (gelatinase A, 72 kDa gelatinase, 72 kDa type IV collagenase)	1.9	0.0387
NM_001075730	STRA6	Stimulated by retinoic acid gene 6	1.9	0.0405
NM_174558	KCNK17	Potassium channel, subfamily K, member 17	1.9	0.0496

**Table 5:** Top 5 functional annotations of up- and down-regulated genes in ICAR of ipsilateral uterine horns

Term	Count	*P*-value
Up-regulated genes		
GO:0008152 ~ metabolic process	38	0.0033
GO:0044237 ~ cellular metabolic process	29	0.0242
GO:0044249 ~ cellular biosynthetic process	15	0.0345
GO:0048878 ~ chemical homeostasis	5	0.0423
Down-regulated genes		
GO:0008152 ~ metabolic process	66	<0.0001
GO:0044237 ~ cellular metabolic process	8	<0.0001
GO:0009987 ~ cellular process	11	0.0001
GO:0044238 ~ primary metabolic process	11	0.0009
GO:0019538 ~ protein metabolic process	23	0.0023

### Gene expression profiles of CAR and ICAR in contralateral uterine horns

A total of 443 and 257 genes were differentially expressed in CAR and ICAR of the contralateral uterine horn of RB cows, respectively when compared with non-RB cows (adjusted *P*-value <0.05, fold-change >1.0). All data of individual gene changes in CAR and ICAR are available in Additional file 1: Tables S3 and S4, respectively. Out of these, 333 genes were up-regulated and 110 genes were down-regulated in CAR, whereas 121 genes were up-regulated and 136 genes were down-regulated in ICAR. The top 10 up- and down-regulated known genes in CAR are shown in Table 6. Similar to CAR of the ipsilateral side, the most pronounced up-regulated gene in RB cows was *GSTA3* (Glutathione *S*-transferase, alpha 3; 12.7-fold). The most down-regulated gene in RB cows was *SLC39A2* (Solute carrier family 39 (zinc transporter), member 2; 2.7-fold). Table 7 shows the top five functional annotations of DEG in CAR between RB and non-RB cows. Biological functions of positive regulation of biological process, positive regulation of cellular process, organ morphogenesis, anatomical structure morphogenesis, and anatomical structure development were highly enriched in up-regulated genes, whereas biological functions of regulation of protein kinase activity, regulation of kinase activity, regulation of transferase activity and carboxylic acid metabolic process were highly enriched in down-regulated genes.

**Table 6:** Top 10 up- and down-regulated known genes in CAR of contralateral uterine horns of RB cows

GenBank accession ID	Gene symbol	Gene description	Fold change	*P*-value
Up-regulated genes				
NM_001077112	GSTA3	Glutathione S-transferase, alpha 3	12.7	0.0080
NM_001014878	PIPOX	Pipecolic acid oxidase	8.4	0.0261
NM_001024569	ELF5	E74-like factor 5 (ets domain transcription factor)	4.3	0.0173
NM_173981	ACAN	Aggrecan	3.0	0.0420
NM_174404	NRXN1	Neurexin 1	3.0	0.0065
NM_001079771	SMOC1	SPARC related modular calcium binding 1	2.7	0.0104
NM_001034351	TNNC1	Troponin C type 1 (slow)	2.6	0.0142
NM_173945	NTS	Neurotensin	2.6	0.0289
NM_001206196	KLHL24	Kelch-like 24 (Drosophila)	2.4	0.0345
NM_001046585	CCL14	Chemokine (C-C motif) ligand 14	2.4	0.0358
Down-regulated genes				
NM_001205648	SLC39A2	Solute carrier family 39 (zinc transporter), member 2	2.7	0.0110
XM_002687754	PLEKHA5	Pleckstrin homology domain containing, family A member 5	2.2	0.0181
NM_001077962	STAC	SH3 and cysteine rich domain	2.0	0.0456
NM_001098061	SQLE	Squalene epoxidase	2.0	0.0268
NM_174145	PKP1	Plakophilin 1 (ectodermal dysplasia/skin fragility syndrome)	1.9	0.0304
NM_001098938	CYP39A1	Cytochrome P450, family 39, subfamily A, polypeptide 1	1.9	0.0262
NM_174489	VLDLR	Very low density lipoprotein receptor	1.9	0.0063
NM_001034660	SLC5A11	Solute carrier family 5 (sodium/glucose cotransporter), member 11	1.8	0.0061
NM_001075803	FH	Fumarate hydratase	1.8	0.0009
NM_001099399	CMTM3	CKLF-like MARVEL transmembrane domain containing 3	1.8	0.0434

**Table 7:** Top 5 functional annotations of up- and down-regulated genes in CAR of contralateral uterine horns

Term	Count	*P*-value
Up-regulated genes		
GO:0048518 ~ positive regulation of biological process	25	<0.0001
GO:0048522 ~ positive regulation of cellular process	22	<0.0001
GO:0009887 ~ organ morphogenesis	12	<0.0001
GO:0009653 ~ anatomical structure morphogenesis	16	0.0001
GO:0048856 ~ anatomical structure development	24	0.0002
Down-regulated genes		
GO:0045859 ~ regulation of protein kinase activity	5	0.0029
GO:0043549 ~ regulation of kinase activity	5	0.0035
GO:0051338 ~ regulation of transferase activity	5	0.0040
GO:0043436 ~ oxoacid metabolic process	7	0.0075
GO:0019752 ~ carboxylic acid metabolic process	7	0.0075

Table 8 shows the top 10 up- and down-regulated known genes in ICAR. The highest increase and decrease in gene expression in RB cows were found for *PIPOX* (Pipecolic acid oxidase; 8.8-fold) and *IFIH1* (Interferon induced with helicase C domain 1; 4.0-fold), respectively. The top five functional annotations of DEG in the ICAR of contralateral uterine horns between RB and non-RB cows are listed in Table 9. The GOs containing genes regulating gene expression, regulation of primary metabolic process, regulation of macromolecule metabolic process, metabolic process and regulation of metabolic process were highly enriched in up-regulated genes. In down-regulated genes, the GO terms involved in primary metabolic process, transport, establishment of localization, localization and metabolic process were highly enriched.

**Table 8:** Top 10 up- and down-regulated known genes in ICAR of contralateral uterine horns of RB cows

GenBank accession ID	Gene symbol	Gene description	Fold change	*P*-value
Up-regulated genes				
NM_001014878	PIPOX	Pipecolic acid oxidase	8.8	0.0156
NM_174278	CNGA1	Cyclic nucleotide gated channel alpha 1	6.8	0.0390
NM_001033608	GSTA3	Glutathione S-transferase, alpha 3	6.6	0.0340
NM_001046400	MIF	Macrophage migration inhibitory factor (glycosylation-inhibiting factor)	3.1	0.0118
NM_001046400	ZNRD1	Zinc ribbon domain containing 1	2.8	0.0400
NM_001206196	KLHL24	Kelch-like 24 (Drosophila)	2.6	0.0212
NM_001076517	LY6D	Lymphocyte antigen 6 complex, locus D	2.5	0.0414
NM_001035473	GK5	Glycerol kinase 5	2.2	0.0210
NM_001075890	KLK10	Kallikrein-related peptidase 10	2.1	0.0445
NM_001083791	SH3BGRL2	SH3 domain binding glutamic acid-rich protein like 2	1.9	0.0030
Down-regulated genes				
XM_002685338	IFIH1	Interferon induced with helicase C domain 1	4.0	0.0485
NM_001101195	KCNA2	Potassium voltage-gated channel, shaker-related subfamily, member 2	3.5	0.0204
NM_180998	LTF	Lactotransferrin	2.9	0.0286
NM_001076843	SLC30A3	Solute carrier family 30 (zinc transporter), member 3	2.6	0.0289
NM_001076494	C8H8orf13	Chromosome 8 open reading frame 13 ortholog	2.5	0.0406
NM_001105411	GFRA1	GDNF family receptor alpha 1	2.5	0.0383
NM_174018	CFTR	Cystic fibrosis transmembrane conductance regulator (ATP-binding cassette sub-family C, member 7)	2.5	0.0316
NM_001077941	MARCH3	Membrane-associated ring finger (C3HC4) 3	2.5	0.0158
NM_173959	SCD	Stearoyl-CoA desaturase (delta-9-desaturase)	2.0	0.0096
NM_174602	SLC2A1	Solute carrier family 2 (facilitated glucose transporter), member 1	1.9	0.0057

**Table 9:** Top 5 functional annotations of up- and down-regulated genes in ICAR of contralateral uterine horns

Term	Count	*P*-value
Up-regulated genes		
GO:0010467 ~ gene expression	17	0.0004
GO:0080090 ~ regulation of primary metabolic process	19	0.0013
GO:0060255 ~ regulation of macromolecule metabolic process	19	0.0015
GO:0008152 ~ metabolic process	38	0.0033
GO:0019222 ~ regulation of metabolic process	19	0.0040
Down-regulated genes		
GO:0044238 ~ primary metabolic process	34	0.0023
GO:0006810 ~ transport	17	0.0025
GO:0051234 ~ establishment of localization	17	0.0026
GO:0051179 ~ localization	18	0.0027
GO:0008152 ~ metabolic process	35	0.0028

### Gene expression profiles of whole uterus

To characterize differential global gene expression profiles in the endometrium of RB and non-RB cows not only locally in each endometrial compartment but also globally in the uterus, we also performed bioinformatics analysis by combining the microarray gene data sets of four endometrial compartments in each cow as whole uterus. A total of 76 genes were found to be differentially expressed in the whole uterus of RB cows when compared with non-RB cows (adjusted *P*-value <0.05, fold-change >2.0). Among these, 37 genes were up-regulated and 39 genes were down-regulated. All up- and down-regulated known genes in the whole uterus are shown in Table 10. The most pronounced up- and down-regulated gene expression in RB cows was found for *PRSS2* (Protease, serine, 2 (trypsin 2); 12.3-fold) and *CHGA* (Chromogranin A (parathyroid secretory protein 1); 3.9-fold), respectively.

**Table 10:** Up- and down-regulated known genes in whole uterus of RB cows as compared with non-RB cows

GenBank accession ID	Gene symbol	Gene description	Fold change	*P*-value
Up-regulated genes				
NM_174690	PRSS2	Protease, serine, 2 (trypsin 2)	12.3	0.0018
NM_001077112	GSTA3	Glutathione S-transferase, alpha 3	6.7	0.0002
NM_001014878	PIPOX	Pipecolic acid oxidase	6.4	<0.0001
NM_174278	CNGA1	Cyclic nucleotide gated channel alpha 1	4.3	0.0024
S82409	IGG1C	IgG1 heavy chain constant region	3.7	0.0081
BC112657	Vl1a	Immunoglobulin lambda light chain variable region	3.7	0.0076
S82407	IgCgamma	IgG2a heavy chain constant region	3.4	0.0347
NM_001025346	DAPL1	death associated protein-like 1	3.4	0.0075
NM_001080353	PI3	Peptidase inhibitor 3, skin-derived (SKALP)	3.2	0.0022
NM_001166616	C5	Complement component 5	2.8	0.0044
NM_001024569	ELF5	E74-like factor 5 (ets domain transcription factor)	2.8	0.0047
NM_001075910	CCDC113	Coiled-coil domain containing 113	2.7	0.0432
NM_173945	NTS	Neurotensin	2.6	<0.0001
NM_001034351	TNNC1	Troponin C type 1 (slow)	2.5	0.0004
NM_001206196	KLHL24	Kelch-like 24 (Drosophila)	2.5	<0.0001
NM_001046400	ZNRD1	zinc ribbon domain containing 1	2.3	<0.0001
NM_001193109	SDCCAG8	Serologically defined colon cancer antigen 8	2.2	0.0001
NM_174010	CD36	CD36 molecule (thrombospondin receptor)	2.2	0.0073
XM_588022	SPOPL	Speckle-type POZ protein-like	2.2	<0.0001
NM_173880	H4	Histone H4	2.1	0.0033
NM_001098155	ZNF322A	Zinc finger protein 322A	2.1	0.0005
NM_001035380	GC	Group-specific component (vitamin D binding protein)	2.0	0.0269
NM_001035473	GK5	Glycerol kinase 5	2.0	0.0003
Down-regulated genes				
NM_181005	CHGA	Chromogranin A (parathyroid secretory protein 1)	3.9	0.0005
NM_001076073	KRT35	Keratin 35	3.3	0.0011
NM_001034728	THBS4	Thrombospondin 4	3.2	<0.0001
NM_001206057	CPXM2	Carboxypeptidase X (M14 family), member 2	3.1	<0.0001
NM_001143735	PRF1	Perforin 1 (pore forming protein)	3.0	0.0090
NM_001002763	CDH1	Cadherin 1, type 1, E-cadherin (epithelial)	2.9	0.0097
NM_176851	FUT5	Fucosyltransferase 5 (alpha (1,3) fucosyltransferase)	2.7	0.0038
XM_002685338	IFIH1	Interferon induced with helicase C domain 1	2.5	0.0040
NM_001081734	MOCS3	Molybdenum cofactor synthesis 3	2.5	0.0465
NM_174039	DPP4	Dipeptidyl-peptidase 4	2.4	0.0158
NM_001102080	CSNK1D	Casein kinase 1, delta	2.3	0.0144
NM_001102060	TBC1D10C	TBC1 domain family, member 10C	2.3	0.0391
NM_001081539	C11H2orf49	Chromosome 11 open reading frame, human C2orf49	2.3	0.0354
AF068848	VpreB	Surrogate light chain	2.3	0.0204
NM_001127317	MIC1	Major histocompatibility class I related protein	2.2	0.0135
NM_205801	CLDN3	Claudin 3	2.2	0.0196
NM_001077887	CLASRP	CLK4-associating serine/arginine rich protein	2.2	0.0245
NM_174513	ADAP1	ArfGAP with dual PH domains 1	2.1	0.0169
NM_001105478	SSLP1	Secreted seminal-vesicle Ly-6 protein 1	2.1	0.0004
NM_001077962	STAC	SH3 and cysteine rich domain	2.1	<0.0001
XM_002687754	PLEKHA5	Pleckstrin homology domain containing, family A member 5	2.1	0.0003
NM_001101239	GRP	Gastrin-releasing peptide	2.1	0.0059
NM_001205648	SLC39A2	Solute carrier family 39 (zinc transporter), member 2	2.0	0.0001

### Validation of gene expression by qPCR

**Fig. 1:**
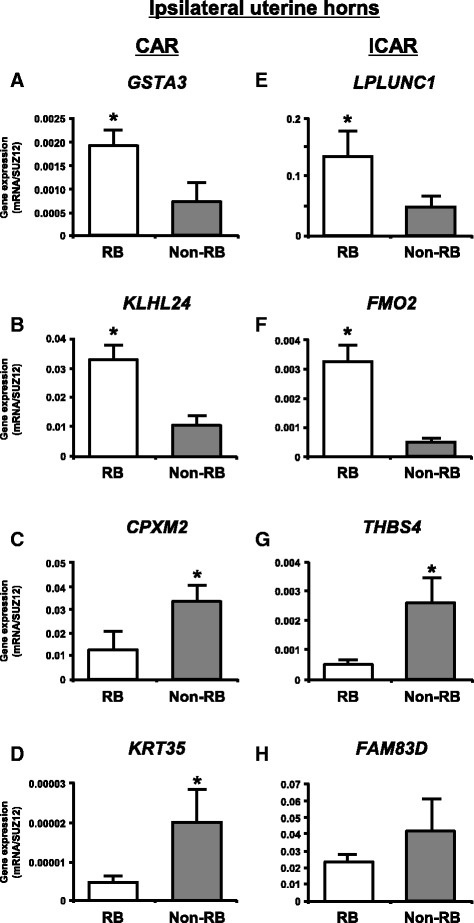
qPCR analysis of top two up- and down-regulated known genes in ipsilateral uterine horns between RB and non-RB cows for validation of the gene expression changes obtained from microarray analysis. **a**, **b**, **c** and **d** CAR and **e**, **f**, **g** and **h** ICAR. **a**, **b**, **e** and **f** up-regulated known genes in RB cows when compared with non-RB cows. **c**, **d**, **g** and **h**) down-regulated known genes in RB cows when compared with non-RB cows. The expression of mRNA was normalized to the expression of *SUZ12* measured in the same RNA preparation. Data are shown as the mean ± SEM. Asterisks show significant differences (*P* < 0.05)

**Fig. 2:**
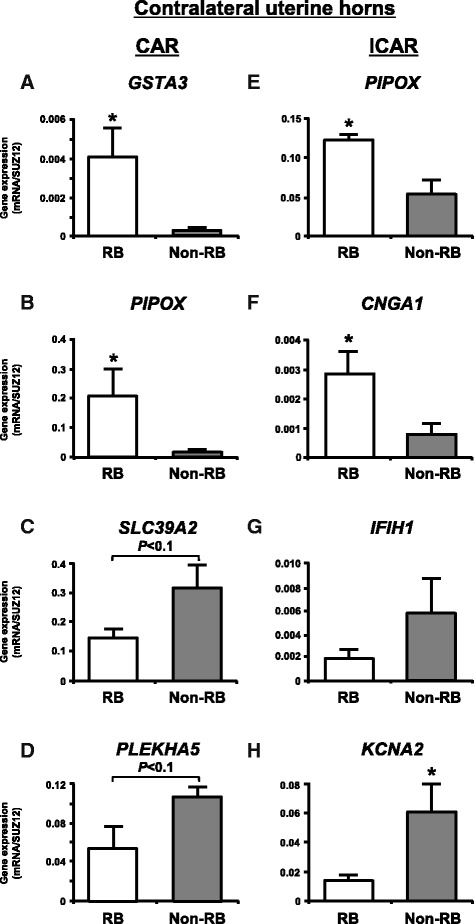
qPCR analysis of top two up- and down-regulated known genes in contralateral uterine horns between RB and non-RB cows for validation of the gene expression changes obtained from microarray analysis. **a**, **b**, **c** and **d** CAR and **e**, **f**, **g** and **h** ICAR. **a**, **b**, **e** and **f** up-regulated known genes in RB cows when compared with non-RB cows. **c**, **d**, **g** and **h** down-regulated known genes in RB cows when compared with non-RB cows. The expression of mRNA was normalized to the expression of *SUZ12* measured in the same RNA preparation. Data are shown as the mean ± SEM. Asterisks show significant differences (*P* < 0.05)

**Fig. 3:**
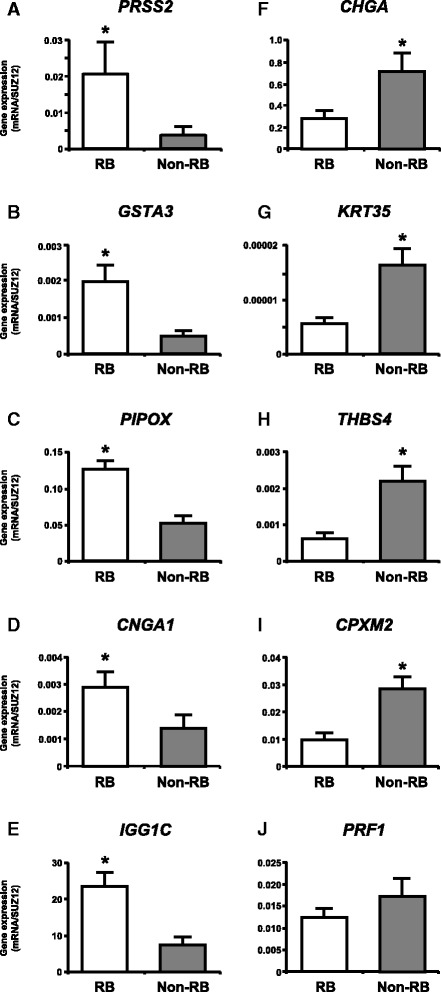
qPCR analysis of top five up- and down-regulated known genes in whole uterus between RB and non-RB cows for validation of the gene expression changes obtained from microarray analysis. **a**, **b**, **c**, **d**, **e** up-regulated known genes in RB cows when compared with non-RB cows. **f**, **g**, **h**, **i**, **j** down-regulated known genes in RB cows when compared with non-RB cows. The expression of mRNA was normalized to the expression of *SUZ12* measured in the same RNA preparation. Data are shown as the mean ± SEM. Asterisks show significant differences (*P* < 0.05)

We selected the top two and top five up- and down-regulated known genes in each endometrial compartment and whole uterus between RB and non-RB cows, respectively to validate the changes in gene expression obtained from microarray analysis by qPCR. qPCR analysis clearly confirmed the microarray results in each endometrial compartment except for *FAM83D* (Fig. 1h), *SLC39A2* (Fig. 2c), *PLEKHA5* (Fig. 2d) and *IFIH1* (Fig. 2g). In the whole uterus, the microarray results were confirmed except for *PRF1* (Fig. 3j).

### Protein localization of CHGA, GSTA3 and PRSS2 in the endometrium of RB and non-RB cows

**Fig. 4:**
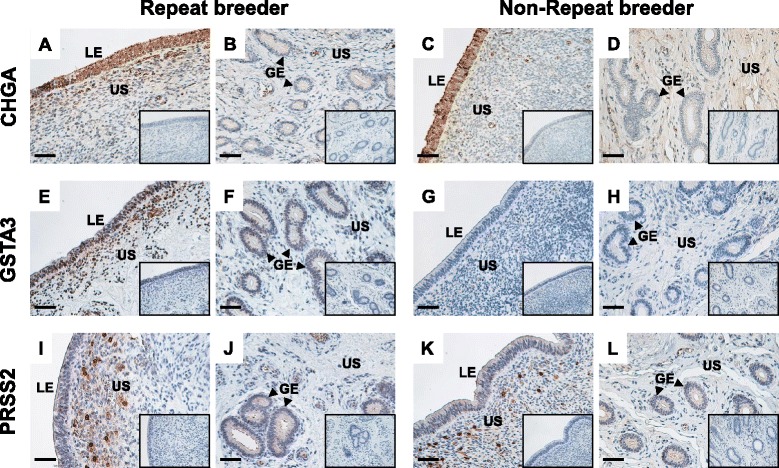
Representative photomicrographs of protein localization of CHGA, GSTA3 and PRSS2 in endometrial tissue from RB and non-RB cows on Day 15 of estrous cycle. Protein localization of (**a**, **b**, **c** and **d**) CHGA, (**e**, **f**, **g** and **h**) GSTA3 and (**i**, **j**, **k** and **l**) PRSS2 in endometrial tissue from RB (**a**, **b**, **e**, **f**, **i** and **j**) and non-RB (**c**, **d**, **g**, **h**, **k** and **l**) cows was detected by immunohistochemistry. Seven-micrometer sections of bovine endometrial tissues of ipsilateral uterine horns on Day 15 of estrous cycle were immunostained with anti-human CHGA, anti-human GSTA3 and anti-bovine PRSS2 polyclonal antibodies. Positive staining of CHGA and PRSS2 were found in the uterine luminal epithelium, uterine stroma and glandular epithelium of both RB and non-RB cows. GSTA3 was detected in the uterine luminal, uterine stroma and glandular epithelium in RB cows, whereas positive staining was not observed in non-RB cows. No signal was detected in the negative control sections using normal rabbit IgG (inserted panels). LE, luminal epithelium; US, uterine stroma; GE, glandular epithelium. Scale bars = 50 μm

Figure 4 shows the results of immunohistochemistry for CHGA, GSTA3 and PRSS2 in the endometrial tissues of ipsilateral uterine horns of RB and non-RB cows on Day 15 of the estrous cycle. In both RB and non-RB cows, a distinct CHGA signal was found in the uterine luminal epithelium and a part of uterine stroma under the epithelium (Fig. 4a and c). CHGA protein was also detected moderately in the glandular epithelium in both RB and non-RB cows and in the uterine stroma in RB cows (Fig. 4b and d). A positive GSTA3 signal was detected in the uterine luminal, uterine stroma and glandular epithelium in RB cows (Fig. 4e and f), whereas positive staining was not observed in non-RB cows (Fig. 4g and h). PRSS2 protein was moderately detected in the uterine luminal epithelium and glandular epithelium, and partially intense staining was observed in the uterine stroma under the epithelium in both RB and non-RB cows (Fig. 4i,j,k and l).

## Discussion

This is the first study to investigate global gene expression profiles of endometrium between RB and non-RB cows in both each endometrial compartments and the whole uterus. As we hypothesized, the microarray analysis identified a number of characteristic up- and down-regulated genes specific to each of four endometrial compartments of RB cows. The RB cows used in this study had experienced pregnancy and then became infertile. Thus, long-term infertility in the RB cows may be associated with alteration of endometrial function. Our results support that alteration of uterine environment, which may be induced by changes in the endometrial gene expression, could be a possible involvement of low fertility in the RB cattle.

Even though the endometrial gene expression profiles were regionally different in the endometrial compartments, *GSTA3* was identified as the most pronounced up-regulated gene in the CAR of both ipsilateral and contralateral uterine horn. GSTA3 is a member of the class Alpha GST isoenzymes which exert a critical role in the detoxification of electrophilic decomposition products generated by reactive oxygen species (ROS) and metabolism of xenobiotics through glutathione conjugation with electrophilic compounds [34–37]. Similar to our results, a recent study has demonstrated that cows with low endometrial receptivity of the embryo show a higher expression of several oxidative stress-response genes in the endometrium compared with highly receptive cows at Day 7 of the estrous cycle [7]. Both oxidative stress and xenobiotics are directly responsible for not only an increase in embryonic mortality but also an alteration of uterine function inducing severe gynecological diseases such as endometriosis and preeclampsia [38–42]. We suppose that the CAR of RB cows may be accompanied by enhanced detoxification and elimination of ROS and xenobiotics. Another important contribution of GSTA3 isomerase is in the biosynthesis of steroids, especially testosterone and P4 in active steroidogenic tissues [43]. Progesterone inhibits endometrial epithelial cell proliferation, adenogenesis and uterine gland development [44, 45]. A previous study showed that RB cows had higher concentrations of P4 receptor in the endometrium than non-RB cows, implying the existence of a local hormonal imbalance in RB cows [46]. In the present study, the *GSTA3* was also highly expressed in the ICAR of RB cows compared with non-RB cows. In addition, immunohistochemistry revealed that a strong signal of GSTA3 protein was detected in the uterine luminal and glandular epithelium and stroma in RB cows. GSTA3 may also be involved in ICAR functions in RB cows by mediating steroidogenesis.

Gene ontology analysis using DAVID revealed that a number of biological processes and functions were different between RB and non-RB cows in both CAR and ICAR. In the CAR of RB cows, genes involved in development and morphogenesis were mainly up-regulated. These genes included 14 and 9 genes regulating embryo development and vasculature development, respectively. The CAR eventually attaches with the trophoblast to give rise to the maternal side of the placentome in pregnant animals [22, 23]. Up-regulation of the genes involved in embryo and vasculature development in the CAR may contribute to the success of implantation and following placental formation at the maternal-fetal interface. An increase in the regulation of these genes in the CAR may be one of the characteristics of the RB uterus. In the ICAR of both the ipsilateral and contralateral uterine horns, genes related to metabolic processes were predominantly enriched in both up- and down-regulated genes in RB cows compared with non-RB cows. The ICAR is a specific compartment containing the uterine glands, which synthesize and secrete various metabolites and histotroph required for estrous cyclicity or development of the conceptus [24]. Alterations of endometrial metabolic processes in RB cows may seriously affect maintenance of uterine function.

The DAVID analysis also revealed that the CAR of the ipsilateral uterine horn of RB cows is characterized by down-regulation of a number of genes associated with cytoskeleton organization, cell adhesion and cellular component organization compared with non-RB cows. Previous global gene expression studies in bovine endometrium showed that profiles of the genes assigned to these functional categories changes during estrous cycle and peri-implantation [11–13], suggesting that these biological functions may be responsible for the regulation of uterine environment. Additionally, the endometrial cell adhesion molecules play a role in conceptus-endometrium attachment at implantation. A direct comparison of cyclic and pregnant endometrium found cell adhesion and cytoskeleton organization molecules affected by pregnancy in both CAR and ICAR [13]. Around the implantation period, the ipsilateral uterine horn is the site of first occurrence of conceptus-endometrial contact and modification of cytological character was seen exclusively on the CAR [47, 48]. Therefore, the lower expression of genes regulating cytoskeleton organization and cell adhesion in CAR of RB cows may be associated with inadequate endometrial responsiveness resulting in implantation failure.

*CPXM2* was included in the top 10 down-regulated genes in both CAR and ICAR of the ipsilateral uterine horn. Previous microarray studies found no differences in *CPXM2* expression in the bovine endometrium between highly fertile and poor fertile, and between highly fertile and subfertile cows at Day 14 of the estrous cycle [9], while expression decreasing at Day 7 compared to Day 3 of estrus in cows with low embryo receptivity [7]. CPXM2 is assumed to be more sensitive to P4 or some CL factors in a poorly fertile endometrium that includes the RB. Although the specific roles of CPXM2 remain unknown, DAVID analysis has assigned it belongs to the biological process of proteolysis and cell adhesion. Thus, *CPXM2* may be related to alteration of endometrial cell adhesion in RB cows, as well as to the above described cell adhesion related genes that are down-regulated in the CAR of the ipsilateral uterine horn of RB cows.

*KLHL24* (Kelch-like 24) was the only gene included in the top 10 up-regulated genes in all four endometrial compartments. A member of the KLHL family including KLHL24 is known to be involved in ubiquitination [49, 50]. It has been reported that lower expression of genes associated with ubiquitination in high fertile as compared with subfertile cows [9]. Although the specific roles of KLHL24 have not yet been elucidated, an increase in oxidative stress stimulated *KLHL24* expression in human fibroblast cells [51], leading us to speculate that this gene is up-regulated to counteract cytoskeleton destruction by ROS- induced cell damage and/or to degrade proteins in cells exposed to ROS by ubiquitination reaction. Therefore, high expression of *KLHL24* in RB cows compared with non-RB cows support the possibility that the endometrium of RB cows is under oxidative stress. However, it has been reported that the level of *KLHL24* gene expression at Day 14 of the estrous cycle shows no significant difference among high fertile, low fertile and infertile cows [9]. The functional contribution of endometrial KLHL24 in bovine fertility remains unclear.

Analysis of the combined gene data sets of the four endometrial compartments revealed gene expression profiles of the whole uterus. *PRSS2* and *CHGA* were the most pronounced up- and down-regulated genes, respectively. PRSS2 is a member of the trypsin family of serine proteases and degrades type I collagen directly or indirectly by activating several procollagenolytic matrix metalloproteinases (MMPs) [52, 53]. CHGA works as a pro-hormone for pancreastatin, vasostatin and catestatin [54–56]. Full-length CHGA and vasostatin act as anti-angiogenic factors to inhibit two potent angiogenic factors, basic fibroblast growth factor (bFGF) and vascular endothelial growth factor, while CHGA cleaved by thrombin and catestatin promote angiogenesis by inducing the release of bFGF from vascular endothelial cells [57]. In the present study, we found that both PRSS2 and CHGA proteins were localized in the luminal and glandular epithelium and in the stroma of the endometrium. These localizations coincide with the tissue site of gelatinase activity of MMP-2 and the localization of MMPs and bFGF in the bovine endometrium [58–60], suggesting paracrine and autocrine actions of PRSS2 and CHGA with MMPs and bFGF in the bovine endometrium. In addition, genes involved in cell death (*DAPL1* and *PRF1*) or cell attachment (*CD36*, *CDH1*, *CPXM2*, *KRT35* and *THBS4*) were also differentially expressed between RB and non-RB cows. Although further studies are needed to clarify, the endometrium of RB cows might not only be involved in the promotion of tissue remodeling and imbalance of angiogenesis but also in the degradation of cell renewal and tissue structure.

In cattle, around Day 15 of pregnancy is a stage of the beginning of conceptus elongation and maternal recognition of pregnancy [26]. A recent RNA-seq study identified numerous conceptus-expressed ligands that interact with corresponding receptors expressed on the endometrium and vice versa at Day 16 of pregnancy in cattle [61]. In the present study, some genes of endometrium expressed ligands (*CCL4*, *CCL14*, *COL1A2*, *EDN1*, *F2*, *MMP2*, *THBS4* and *TIMP3*) and receptors (*ACVR2B*, *BMPR2*, *CD4*, *CD36*, *IGF2R*, *IL10RB*, *KDR*, *TNFRSF25* and *VLDLR*) that interact with conceptus reported by Mamo et al. were differentially expressed between RB and non-RB cows. In addition, other genes encoding growth factors (*FGF9* and *GDF7*) and cytokines (*CCL8*, *CD14* and *CD53*) were down-regulated in the RB cows as compared with non-RB cows. Although the functional role of these two growth factors in bovine endometrium remains to be elucidated, FGF9 induces endometrial stromal cell proliferation [62]. Up-regulation of *FGF9* and *GDF7* expressions were detected in equine and/or swine pregnant endometrium and may be implicated in embryo-maternal communication at early pregnancy [63, 64]. The receptors of these growth factors were expressed in not only endometrium but also conceptus at Day 16 of pregnancy in cattle [61]. Therefore, alteration of the expression of these ligands and receptors in the RB cows may affect conceptus development and maternal recognition of pregnancy if a conceptus presents in the RB cows.

## Conclusion

The results of the present study support the hypothesis that endometrial gene expression profiles are different between RB and non-RB cows. In RB cows, characteristic gene expression was identified in both the CAR and ICAR of both ipsilateral and contralateral uterine horns. The enriched GO terms of these genes were related to cell adhesion and morphogenesis in the CAR and metabolism in the ICAR. These results suggest that local regulation of molecular mechanisms in each endometrial compartment may contribute to normal uterine physiology. Therefore, the identified candidate endometrial genes and functions are likely to be involved in bovine reproductive performance. The present study could provide an information base for understanding underlying molecular pathogenesis and developing a treatment of repeat breeding in cattle from the point of view of endometrial function.

## Additional file

Additional file 1:
**Table S1.** List of up- and down-regulated genes in CAR of ipsilateral uterine horns of RB cows (*n* = 4) on Day 15 of the estrous cycle as compared with non-RB cows (*n* = 4). **Table S2.** List of up- and down-regulated genes in ICAR of ipsilateral uterine horns of RB cows on Day 15 of the estrous cycle as compared with non-RB cows. **Table S3.** List of up- and down-regulated genes in CAR of contralateral uterine horns of RB cows on Day 15 of the estrous cycle as compared with non-RB cows. **Table S4.** List of up- and down-regulated genes in ICAR of contralateral uterine horns of RB cows on Day 15 of the estrous cycle as compared with non-RB cows. (XLSX 254 kb)

## Abbreviations

ACVR2B: Activin A receptor type 2B
AI: Artificial insemination
bFGF: Basic fibroblast growth factor
BMPR2: Bone morphogenetic protein receptor type 2
CAR: Caruncular
CCL: Chemokine (C-C motif) ligand
CD4: CD4 molecule
CD14: CD14 molecule
CD36: CD36 molecule
CD53: CD53 molecule
CDH1: Cadherin 1, type 1
CHGA: Chromogranin A
CL: Corpus luteum
COL1A2: Collagen type I alpha 2 chain
CPXM2: Carboxypeptidase X (M14 family), member 2
DAPL1: Death associated protein-like 1
DAVID: Database for annotation, visualization and integrated discovery
DEG: Differentially expressed genes
EDN1: Endothelin 1
F2: Coagulation factor II, thrombin
FAM83D: Protein FAM83D
FGF9: Fibroblast growth factor 9
GDF7: Growth differentiation factor 7
GE: Glandular epithelium
GEO: Gene expression omnibus
GO: Gene ontology
GSTA3: Glutathione S-transferase A3
ICAR: Intercaruncular
IFIH1: Interferon induced with helicase C domain 1
IGF2R: Insulin like growth factor 2 receptor
IL10RB: Interleukin 10 receptor subunit beta
KDR: Kinase insert domain receptor
KLHL24: Kelch-like 24
KRT35: Keratin 35
LE: Luminal epithelium
LPLUNC1: Von Ebner minor salivary gland protein
MMP: Matrix metalloproteinase
P4: Progesterone
PIPOX: Pipecolic acid oxidase
PLEKHA5: Pleckstrin homology domain containing, family A member 5
PRF1: Perforin 1
PRSS2: Trypsin 2
qPCR: Quantitative real-time RT-PCR
RB: Repeat breeder
ROS: Reactive oxygen species
SLC39A2: Solute carrier family 39 (zinc transporter), member 2
SUZ12: Suppressor of zeste 12
THBS4: Thrombospondin 4
TIMP: Tissue inhibitor of metalloproteinase
TNFRSF25: Tumor necrosis factor receptor superfamily member 25
US: Uterine stroma
VLDLR: Very low density lipoprotein receptor
